# Areas of Work Life as Predictors of Occupational Burnout of Nurses and Doctors in Operating Theaters in Poland—Multicenter Studies

**DOI:** 10.3390/healthcare10010026

**Published:** 2021-12-24

**Authors:** Piotr Jarzynkowski, Renata Piotrkowska, Wioletta Mędrzycka-Dąbrowska, Janina Książek

**Affiliations:** 1 Department of Surgical Nursing, Faculty of Health Sciences, Medical University of Gdansk, 80-211 Gdańsk, Poland; piotr.jarzynkowski@gumed.edu.pl (P.J.); renata.piotrkowska@gumed.edu.pl (R.P.); janina.ksiazek@gumed.edu.pl (J.K.); 2 Department of Anaesthesiology Nursing & Intensive Care, Faculty of Health Sciences, Medical University of Gdansk, 80-211 Gdańsk, Poland

**Keywords:** burnout, operating theater, nurses, doctors

## Abstract

Introduction: Researchers’ interest in occupational burnout results primarily from the dangerous and extensive consequences of this phenomenon. The aim of the study was to analyze the level of occupational burnout among nurses and doctors in operating theaters. Materials and Methods: A cross-sectional survey study conducted on 325 nurses and doctors of seven hospitals in Poland. The Maslach Burnout Inventory (MBI) and the Areas of Worklife Survey (AWS) by Michael Leiter and Christina Maslach. Results: The mean values for the level of occupational burnout for the entire sample according to the scale from the Maslach Burnout Inventory by C. Maslach amounted to 14.35 for emotional exhaustion, 8.56 for depersonalization, and 11.90 for personal accomplishment; when compared to reference levels, they classified emotional exhaustion at a low level, depersonalization at an average level, and personal accomplishment at a high level of burnout. Areas of work life are predictors of occupational burnout. The analysis showed a relationship between three of the six variables. As the workload increased, so did the level of burnout among participants, and the categories of honesty and values. Conclusions: The conducted research has shown that occupational burnout among nurses and doctors in operating theaters occurs in all dimensions of this phenomenon (emotional exhaustion, depersonalization, job satisfaction). It was also shown that the areas of work life (workload, control, community, rewards, fairness, values) are predictors of occupational burnout among the respondents. This article shows how important the problem of burnout among operating theater medical staff is. Perhaps it will allow nurses and doctors to recognize this syndrome and encourage them make changes to their work to prevent burnout.

## 1. Introduction

Researchers’ interest in occupational burnout results primarily from the dangerous and extensive consequences of this phenomenon. Its health-related, mental and social effects affect not only the employees themselves, but also their work and family environment [1,2].

An important contribution is that of Freudenberger, who in his article used the words Staff Burnout to denote the exhaustion of an individual caused by the overload of tasks assigned to their social and physical work. Thus, the researcher introduced the term “burnout” to scientific vocabulary, defining it as “a drop in an employee’s energy, emerging as a result of being overburdened by the problems of others” [3].

A slightly different, multidimensional definition of occupational burnout was provided by Ch. Maslach, who described it as “a psychological syndrome of emotional exhaustion, depersonalization and a lowered sense of personal accomplishment that can be observed in people working with others in a certain specific manner” [4]. After research carried out on psychiatrists, social care staff and prison service staff, Ch. Maslach and S. Jackson described burnout as a long-term reaction to chronic interpersonal and emotional stressors appearing in people working in close contact with others [5].

This definition accentuates that burnout syndrome is largely related to the work of people whose jobs involve care for others, such as medical staff. The operating theater is a very special space in the hospital structure. It is a hermetic room which can be accessed solely by its staff and patients scheduled for an operation at a given time [6,7]. The staff of the hospital operating theatre can be divided into two basic groups. The first, group one, is its regular staff, including operating room nurses and nurse anesthesiologists, anesthesiologists, and medical support staff. The second is temporary staff, including surgeons, trainees, students, as well as participants of various training or specialty courses [8]. Work in an operating theater requires, above all, a team effort of many people. Staff members must not only have high professional qualifications and keep broadening their knowledge concerning progress in medicine, but also possess the knowledge and skills in the area of the operation of highly specialized medical devices and equipment [9].

The burdens resulting from such work may be both mental and physical. The mental burdens result mainly from responsibility for the patient’s health and life. This is amplified by the necessity to simultaneously cooperate with a team of surgeons and anesthesiologists. Particularly burdening is providing assistance in operations on patients with multiorgan injuries, organ transplantations, and in the case of the patient’s death during surgery [10]. The physical burdens include assisting in operations lasting for many hours, the related enforced body position, the lifting and transporting of patients, carrying sets of surgical tools, the specific microclimate of the operating room, and the noise of the equipment [11]. These burdens are also connected to contact with anesthetic agents, exposure to X-ray and laser radiation, as well as magnetic fields and high voltage electricity. Additionally, the chemicals used for disinfection, gloves, talc, and other substances used for the fixation of preparations have an irritating effect on the skin. Direct contact with infectious materials (blood, body fluids, human waste, bone shavings) is related to a high risk of infection. Work in operating theaters is therefore challenging and requires good health, physical fitness, and resistance to stress. A well-organized and well-equipped operating theater, qualified medical staff, good cooperation in the team, a friendly atmosphere, as well as observation of procedures and standards lower the risk of physical and mental overload, resulting in nurses and doctors working in operating theaters being less susceptible to stress and, in consequence, to occupational burnout [12].

There is a lot of research and reports on burnout among medical professionals, but few about operating theaters (nurses and doctors). The aim of the study was to analyze the level of occupational burnout among nurses and doctors in operating theaters.

## 2. Methods

### 2.1. Design

Multicenter cross-sectional survey study.

### 2.2. Study Population

Participants in this study were 325 people (221 nurses and 114 doctors) from seven hospitals in the Pomorskie and Kujawsko-Pomorskie provinces, because it is the first phase of a major research project. In the next stages, tests will be carried out in operating theaters of hospitals in other Polish provinces. The research was conducted between March 2018 and April 2019. It was decided to conduct research in these hospitals because these hospitals have highly specialized operating theaters, where a large number of surgeries are performed and a large number of highly qualified medical personnel is employed. Eligibility criteria included work experience in an operating theater of more than one year and performing the job of a nurse or a doctor.

### 2.3. Data Collection

Survey questionnaires were handed out to the participants during visits to the operational theaters in which they were employed. Participation was voluntary and anonymous. All participants expressed their consent to participate and were informed about the aim and procedure of the study.

### 2.4. Questionnaire Development

A diagnostic survey method was applied. The survey was based on standardized research tools: the Maslach Burnout Inventory (MBI) and the Areas of Worklife Survey (AWS) by Christina Maslach and Michael Leiter. Demographic details were also collected concerning gender, education, and years in the workforce.

#### 2.4.1. The Maslach Burnout Inventory (MBI)

The Maslach Burnout Inventory was developed in 1981 by Christina Maslach and Susan Jackson [13]. In this study, its Polish adaptation by Pasikowski was used for the measurement of three elements of the participants’ occupational burnout syndrome: emotional exhaustion, depersonalization, and personal accomplishment [14]. In terms of psychometric properties, it is a very valuable and most frequently used tool designed for the examination of the level of occupational burnout. The questionnaire consists of a total of 22 items divided into three groups—each of them concerning one of the elements of burnout syndrome. The results were calculated separately for each of the subscales. According to the authors, the Maslach Burnout Inventory can be used among occupational groups whose work consists of having specific contact with others. Table 1 shows the reference MBI levels for healthcare professionals [13].

#### 2.4.2. Areas of Worklife Survey by Ch. Maslach and M. Leiter (AWS)

The Areas of Worklife Survey by Christina Maslach and Michael Leiter in its Polish adaptation is by Jan F. Terelak et al. was used [15]. This method is used for the assessment of the functioning of employees in their work setting in their particular areas and enables the identification of inconsistencies between the organization’s requirements and the participants’ aspirations, possibilities, and needs. It relates to six areas of work life, which in certain specific conditions have an impact on the future development of occupational burnout. These include: 1. workload—sense of being loaded with work: whether one considers that they can tackle their situation at work and the tasks entrusted to them, or whether they feel overwhelmed or overburdened by excessive work; 2. control—allows to assess one’s possibility to take independent decisions, make choices independently of others; 3. rewards—concerns one’s degree of satisfaction with rewards they obtain for their work: both material rewards and opportunities for promotion, as well as social ones, such as recognition and respect from colleagues, superiors, and clients; 4. community—concerns evaluation of the quality of the social environment at the workplace, i.e., one’s sense of mutual support, cooperation, and mutual expression of positive feelings by team members; 5. fairness—applies to the employee’s sense of whether they are treated in a just way and concerns aspects of work such as clear rules, the distribution of goods, and opportunities for promotion; 6. values—allows to assess whether there is a conflict of values within the organization or a conflict between the employee’s values and the values supported and promoted by the organization.

### 2.5. Ethical Considerations

The study was approved by the Independent Bioethical Committee for Scientific Research of the Medical University of Gdańsk (No. NKBBN/51//2018).

### 2.6. Statistical Analysis

All statistical calculations were carried out with the help of the statistical package IBM SPSS 23 and an Excel 2013 spreadsheet. Qualitative variables were presented as counts and percentages, while quantitative variables were described with the help of the arithmetic mean and standard deviation. To check whether quantitative variables came from a normally distributed population, the Shapiro–Wilk test was used. The Mann–Whitney U test and Student’s *t*-test were used to check the significance of differences between two groups, while the Kruskal–Wallis test was applied to check the significance between more than two groups. To predict and check the impact of several factors (independent variables) on dependent variables, multiple linear regression analysis was carried out. To determine the dependence between the strength and direction of variables, correlation analysis was performed by calculating Spearman’s rank correlation coefficients. In all calculations, the level of significance was set at *p* < 0.05.

## 3. Results

### 3.1. Sociodemographic Characteristics

The sample included many more women (74.2%) than men (25.8%). As far as their marital status was concerned, more than 60% of the participants were in a relationship. The study was carried out in two provinces: 68% of respondents worked in the Pomorskie Province, while 32% of them in the Kujawsko-Pomorskie Province. Their level of education was significant. Most of the participants (32.3%) were medical faculty graduates, while 23.1% had graduated from a secondary nursing school. On average, every fifth respondent (20.9%) had a bachelor’s degree in nursing, and a further 8.3% had a master’s degree in nursing. The participants’ age range was broad. On the date of the study, the youngest respondent was 23 years old, while the oldest one was 63 years old. Among the participants, 45.2% were operating room nurses. On average, every fifth respondent (19.7%) was a nurse anesthesiologist. There were also surgeons (13.2%) and anesthesiologists (11.4%).

The number of years worked by the respondents ranged from 1.5 to 43 years (M = 20.91; SD = 10.2). More than half of the sample (58.8%) worked as a part of a 12 h shift system, while 41.2% as a part of a single 8 h shift system. The study discovered a statistically significantly higher occupational burnout rate for women than for men (Z = −2.28; *p* < 0.05).

To check whether the participants’ education had an impact on their occupational burnout, the Kruskal–Wallis test was used. The analysis showed a relationship between variables (H(7) = 23.15; *p* < 0.05). To find out to which groups it applied, Bonferroni’s correction was used. It was discovered that a statistically more significant burnout rate applied to the respondents with a bachelor’s degree in nursing in comparison with the respondents who graduated from a medical faculty. No statistically significant relationship (*p* > 0.005) was discovered between the other groups. In yet another part of the study it was shown that a statistically significantly higher occupational burnout rate applied to operating room nurses than to surgeons and orthopedists. Other sociodemographic factors such as age, marital status, number of years worked, place of residence, and shift work did not affect the participants’ level of burnout.

### 3.2. Occupational Burnout according to the Maslach Burnout Inventory (MBI) Questionnaire

The mean values of occupational burnout dimensions for the participants working as nurses and doctors according to the Maslach Burnout Inventory (MBI) in comparison with reference values for healthcare professionals classified emotional exhaustion at a low level, depersonalization at an average value, and personal accomplishment at a high level of occupational burnout (Table 2).

### 3.3. Professional Burnout according to the Areas of Worklife Survey (AWS) Questionnaire

The subsequent part of the study checked the relationship between the level of the participants’ occupational burnout and the following areas of their work life: workload, control, community, rewards, fairness, and values. The analysis showed a relationship between three of the six variables. The level of occupational burnout among the participants grew along with an increase in their workload and the categories of fairness and values (Table 3).

### 3.4. Areas of Work Life as Predictors of Emotional Exhaustion, Depersonalization, and Personal Accomplishment

In this part of the study, we examined whether certain areas of work life are predictors of the respondents’ emotional exhaustion, depersonalization, and professional satisfaction. Regression analysis showed a significant prediction of the level of emotional exhaustion with the help of selected predictors (F(6.318) = 15.41; *p* < 0.001). Analysis demonstrated that the correlation between areas of work life and emotional exhaustion was moderate. The multiple correlation coefficient amounted to R = 0.47. An analysis of the corrected R-squared showed that areas of work life explained about 21% of the variability of emotional exhaustion. The corrected R-squared amounted to 0.21. Significance tests indicated a relationship between emotional exhaustion and two variables: workload and values (Table 4).

A detailed analysis of the unstandardized coefficients (B) demonstrated that along with a one measuring point increase in workload, the level of emotional exhaustion grew by 0.23 of the measuring point (B = 0.23), and along with an increase in values, the level of emotional exhaustion increased by 0.18 of the measuring point (B = 0.18). Regression analysis showed a significant prediction of the level of depersonalization with the help of selected predictors (F(6.318) = 6.96; *p* < 0.001). Analysis demonstrated that the correlation between areas of work life and depersonalization was moderate. The multiple correlation coefficient amounted to R = 0.34, while an analysis of the corrected R-squared showed that areas of workload explained about 9% of the variability of depersonalization. The corrected R-squared amounted to 0.09. Significance tests indicated a relationship between the respondent’s depersonalization and three variables: workload, rewards, and fairness (Table 5).

A detailed analysis of the unstandardized coefficients (B) showed that along with a one measuring point increase in workload, depersonalization increased by 0.07 of the measuring point (B = 0.07), and that along with an increase in rewards, depersonalization decreased by 0.15 of the measuring point (B = 0.15), while along with an increase in fairness, depersonalization increased by 0.06 of the measuring point (B = 0.06). Regression analysis showed a significant prediction of the level of personal accomplishment through selected predictors (F(6.318) = 39.22; *p* < 0.001). Analysis demonstrated that the correlation between areas of work life and personal accomplishment was moderate. The multiple correlation coefficient was R = 0.39, while an analysis of the corrected R-squared showed that areas of work life explained about 14% of the variability of personal accomplishment. The corrected R-squared amounted to 0.14. Significance tests indicated a relationship between personal accomplishment and two variables: workload and control (Table 6).

A detailed analysis of the unstandardized coefficients (B) revealed that along with a one measuring point increase in workload, the level of personal accomplishment decreased by 0.08 of the measuring point (B = 0.08), and that along with an increase in control, the level of personal accomplishment decreased by 0.19 of the measuring point (B = 0.19).

## 4. Discussion

Occupational burnout syndrome has been described in relevant literature for more than 40 years. Since 1970, more than 5500 studies and books devoted to the topic have been published, and the number is increasing on an annual basis [16]. This shows how important this phenomenon is [17]. Numerous studies demonstrate that burnout is more often experienced by representatives of professions which deal with the provision of social services, require close, direct work with others and personal involvement in interpersonal relations, which are often connected with the provision of support to people, and in which social skills are a fundamental work tool [18]. This is confirmed in Molina-Praena’s research, in which 31% of nurses were diagnosed with emotional exhaustion, 24% with high depersonalization, and 38% with low personal accomplishment [19], and in Ramuszewicz’s study, in which 32% of the operating room nurses involved described themselves as experiencing occupational burnout, and almost half of them (42%) felt stressed [20].

These findings have also been partially confirmed in our own research, which showed that a statistically significantly higher degree of occupational burnout was experienced by operating room nurses than by surgeons and orthopedists. Among persons working in operating theaters, occupational burnout is not often selected as a topic of studies. Most often, the studies are conducted on people performing specific tasks related to their own specialty in the operating theater. Wojciechowska et al. focused on operating room nurses. They demonstrated that nurses employed in the operating theaters are much more exposed to the risk of occupational burnout syndrome. At the same time, they recorded a certain relationship between the work setting and the degree of occupational burnout [21].

Norwegians adopted another research attitude to the phenomenon of occupational burnout—they performed their study on doctors and nurses working in operating theaters and surgical wards in 15 national hospitals. There were 2601 participants in this study (2050 nurses and 551 doctors). In their results, they specified the elements giving employees satisfaction and simultaneously preventing occupational burnout. Among the doctors, it was mainly cooperation and a dominating position. For the nurses, it was the atmosphere in the team based on partnership, including recognition of their rights as equal with the doctors’ rights. These studies have shown the importance of good cooperation between doctors and nurses in order to prevent burnout and lead to an improvement in quality and hence patient safety [22].

It results from our own research that only some sociodemographic factors are related to the participants’ occupational burnout. One of them is gender. Women suffered from a higher level of occupational burnout than men. This conclusion was presented in studies by researchers from Belgium, Germany, USA, and Canada [7].

During the subsequent stage of our research, it was demonstrated that the occupational burnout rate was statistically significantly higher in participants with a bachelor’s degree in nursing than in graduates of six-year studies in medicine. This confirms the higher occupational burnout rate in operating room nurses than in surgeons and orthopedists. Hallsten et al. indicated an impact of higher education on the level of occupational burnout and established that higher education is most often related to greater responsibility, and sometimes also a greater scope of duties. However, it cannot be considered a predictor of occupational burnout [23].

Other researchers demonstrated a difference of the level of occupational burnout depending on one’s medical qualifications, indicating that among five medical professions, the highest level of occupational burnout was experienced by nurses (66%), with physician’s assistants (61.8%), doctors (38.6%), administrative staff (36.1%), and medical technicians (31.9%) affected less [24]. Among the studied sociodemographic factors, the respondents’ age, place of residence, marital status, number of years worked, and shift work had no impact on the level of occupational burnout. The first three factors do not play a significant role in the majority of studies by other authors, although some studies indicate a statistically significant relationship. Examples include research by Włodarczyk et al., who performed research on nurses working in operating theaters. The authors showed that among the demographic factors, place of residence and gender play a significant role. One’s residence in a city and the male gender were related to a lower degree of the absence of involvement. Residence in a city was also related to an expected lower rate of exhaustion [2].

The authors stressed that what seemed significant here was the distance between one’s place of work and one’s place of residence: the greater this was, the higher the dissatisfaction and the faster the exhaustion. Our own research did not demonstrate such a relationship, although the participants lived in places situated at various distances from their place of work. Similar results were generated in the case of marital status—no relationship was discovered between the variables. However, other researchers have confirmed such a relationship in their studies—they demonstrated that non-married individuals were exposed to a higher risk of the absence of involvement in their work than married people, with a greater risk of exhaustion in divorced participants than in married ones [25,26]. These results seem to be consistent with the theory on social support from one’s family as a resource protecting against the negative consequences of stress at work.

Our own research did not show a relationship between the respondents’ age and the number of years worked and burnout. There are studies, however, showing that these variables have an impact on the level of occupational burnout. For example, Hatch et al. demonstrated that nurses with a higher number of years worked display a greater level of occupational burnout [27].

Areas to be analyzed include shift work, which, according to the generated results, had no statistically significant impact on the respondents’ level of occupational burnout. Researchers demonstrated a negative impact of shift work on the level of occupational burnout from the point of view of there being an excessive burden on the balance between the quality of one’s personal and professional life as early as in 2013 [28]. Many researchers consider shift work and, above all, its rhythm prolonged to 12 h, a factor facilitating the development of occupational burnout syndrome, and even as a stressor [29]. Other researchers showed that nurses working on a full-time basis report a higher level of occupational burnout than nurses working a on a part-time basis [30]. This is also confirmed by research devoted to flexible working time, which clearly demonstrated that the possibility to control one’s working time allows a decrease in occupational burnout [31,32].

The results generated in the group of nurses and doctors of operating theaters under this study allow to conclude that people who were more burdened with work displayed a higher level of occupational burnout. Overburdening with occupational work may result both from the excessive responsibility related to work in the operating theater, but also from undertaking too many working hours. Studies by other authors show that a high level of responsibility of employees of hospital operating theaters is related to the risk of medical errors and has an impact on the level of occupational burnout [33].

Emotional burnout is marked by specific symptoms, including helplessness, lack of energy, weakness, fatigue, irritability, and proneness to conflicts [34]. Our research showed a significant prediction of the level of emotional exhaustion through selected predictors (workload and values). Some studies show that emotional exhaustion is strongly related to pressure at work, which is very high in the staff of operating theaters [10]. The staff of operating theaters is regularly exposed to many factors causing stress at work (shortages of staff and equipment, the necessity to supervise employees having less experience), which calls for more effort to be able to cope with the daily professional challenges. In consequence, they gradually develop the sense of weakening and occupational burnout [35].

Depersonalization is most often manifested in the treatment of others as objects, cynicism, indifference, routine, treatment of the patient as yet another case, a change of care into supervision, and avoidance of contact with patients and their families [36]. Our research shows that depersonalization concerns employees of operating theaters to a medium degree. When lowered, the third dimension of occupational burnout, referred to as personal accomplishment, leads to self-perception as an ineffective and incompetent individual, the sense of absence of achievements and successes, as well as the loss of the sense of work. It also manifests itself in the worsening of health, increased absence from work, negative emotions, and conflicts. In consequence, the quality of services provided by medical staff is lowered [37].

An analysis of our research demonstrated that the correlation between areas of work life and the level of professional satisfaction was moderate. Similar results were obtained in studies conducted on nurses in Spain and Germany [38,39], where nurses indicated the absence of balance between their workload and rewards, e.g., absence of promotion (absence of the sense of personal accomplishment) as one of the main factors leading to occupational burnout.

## 5. Limitations

The above research is limited as it was carried out only in several hospitals in Poland, on a relatively small number of nurses and doctors. Hence, we are risking a generalization of the results to all the operating room nurses and nurse anesthesiologists, anesthesiologists, and surgeons of various specialties working in operating theaters all over Poland. For this reason, this study should be repeated in many centers on a larger sample. This will allow an identification of the prevalence and reasons behind the occupational burnout among staff of operating theaters in Poland and, in consequence, the introduction of preventive and assistance programs aimed at the prevalence of this syndrome.

## 6. Conclusions

The conducted research has shown that occupational burnout among nurses and doctors in operating theaters occurs in all dimensions of this phenomenon (emotional exhaustion, depersonalization, job satisfaction). It was also shown that areas of work life (workload, control, community, rewards, fairness, values) were predictors of occupational burnout among the respondents.

## 7. Implication for Practice

The presented research shows that occupational burnout of nurses and doctors of operating theaters is a considerable problem. In order to prevent the phenomenon, we need to undertake broad preventive measures. A particularly important role in this task should be played by employers through the planning and delivery of training events aimed at self-diagnosis of the symptoms of occupational burnout and education in the field of techniques and methods of coping with it. The organization should aim at the improvement of the work setting through better communication and the prevention of conflicts. Other important factors include fair management, the introduction of flexible working plans, and the precise determination of the professional duties and observation of rules, norms, procedures, and standards in place in a given facility. In the case of operating theaters, the technical condition of medical devices and equipment should be checked out on a regular basis, and personal protective equipment should be used. Nurses and doctors could be assessed for burnout and offered professional help if necessary. It also seems indispensable to introduce a subject related to the occupational burnout and stress of medical personnel to professional education at the level of both medical and nursing studies. All these actions are realistic and feasible. They will allow for an increase in work satisfaction among nurses and doctors working in operating theaters, thus increasing the quality of the provided care.

## Figures and Tables

**Table 1:** Reference levels of professional burnout according to MBI for health care workers.

Reference Levels	Proffesional Burnout Dimension		
	Emotional Exhaustion	Depersonalizaton	Pesonal Accomplishment
High	27 or more	10 or more	33 or less
Medium	19–26	6–9	39–34
Low	18 or less	5 or less	40 or more

**Table 2:** Mean values of the occupational burnout level for the group under study according to the MBI questionnaire.

Elements of Occupational Burnout	*N*	*Min*	*Max*	*M*	*SD*
Emotional exhaustion	325	8	18	14.35	2.74
Depersonalization	325	4	10	8.56	1.53
Personal accomplishment	325	8	16	11.90	2.17

*M*—mean; *SD*—standard deviation.

**Table 3:** Areas of work life and occupational burnout.

Areas of Work Life	*N*	*rHO*	*p*
Workload	325	0.28	<0.001
Control	325	0.04	0.450
Community	325	0.09	0.115
Rewards	325	0.04	0.505
Fairness	325	0.14	0.011
Values	325	0.16	0.005

*N*—number of respondents, *rHO*—Spearman’s rank correlation coefficient, *p*—significance level.

**Table 4:** Emotional exhaustion and areas of work life.

Model	Unstandardized Coefficients		Standardized Coefficients	*t*	Significance
	B	Standard Error	Beta		
Constant	6.854	1.152	-	5.949	<0.001
Workload	0.233	0.033	0.381	7.060	<0.001
Control	−0.016	0.068	−0.014	−0.240	0.811
Rewards	−0.062	0.087	−0.041	−0.711	0.478
Community	0.062	0.047	0.076	1.320	0.188
Fairness	0.053	0.040	0.079	1.345	0.180
Values	0.176	0.057	0.167	3.091	0.002

*t*—result of Student’s *t*-test.

**Table 5:** Depersonalization and areas of work life.

Model	Unstandardized Coefficients		Standardized Coefficients	*t*	Significance
	B	Standard Error	Beta		
Constant	6.937	0.687	-	10.103	<0.001
Workload	0.077	0.020	0.228	3.946	<0.001
Control	0.043	0.040	0.066	1.060	0.290
Rewards	−0.155	0.052	−0.182	−2.990	0.003
Community	0.010	0.028	0.022	0.352	0.725
Fairness	0.063	0.024	0.167	2.670	0.008
Values	0.034	0.034	0.058	1.006	0.315

*t*—result of Student’s *t*-test.

**Table 6:** Personal accomplishment and areas of work life.

Model	Unstandardized Coefficients		Standardized Coefficients	*t*	Significance
	B	Standard Error	Beta		
Constant	17.040	0.952	-	17.897	<0.001
Workload	−0.082	0.027	−0.170	−3.020	0.003
Control	−0.195	0.056	−0.211	−3.472	0.001
Rewards	0.030	0.072	0.025	0.423	0.673
Community	−0.044	0.039	−0.069	1.154	0.249
Fairness	−0.039	0.033	−0.073	1.194	0.233
Values	−0.049	0.047	−0.059	1.048	0.295

*t*—result of Student’s *t*-test.

## Data Availability

The authors declare that the data of this research are available from the correspondence author upon request.
